# Body Image Quality of Life Related to Light Physical Activity and Sedentary Behavior among Young Adults with Overweight/Obesity

**DOI:** 10.3390/bs11080111

**Published:** 2021-08-12

**Authors:** Jamie M. Faro, Jessica A. Whiteley, Laura L. Hayman, Melissa A. Napolitano

**Affiliations:** 1 Department of Population and Quantitative Health Sciences, University of Massachusetts Medical School, Worcester, MA 01605, USA; 2 Department of Exercise and Health Sciences, University of Massachusetts Boston, Boston, MA 02125, USA; Jessica.Whiteley@umb.edu; 3 Department of Nursing, University of Massachusetts Boston, Boston, MA 02125, USA; Laura.hayman@umb.edu; 4 Department of Prevention and Community Health, The George Washington University, Washington, DC 20052, USA; mnapolitano@gwu.edu; 5 Department of Exercise and Nutrition Sciences, Milken Institute School of Public Health, Washington, DC 20052, USA

**Keywords:** physical activity, body image quality of life, sedentary, weight loss

## Abstract

Sedentary behaviors, low levels of physical activity (PA), and low body image quality of life have been identified during college years and associated with poor health outcomes. Public health efforts have recently focused on decreasing sedentary time by increasing light physical activity, both of which have been associated with body image quality of life, though mainly through self-report. In this cross-sectional study, we examined objective actigraphy and survey data from 404 of 459 young adults with overweight and obesity (mean age 23.3 ± 4.4 years, 78.4% female, 55.4% white). PA was measured using an accelerometer worn during waking hours for >10 h/day for four days. Body image quality of life was assessed using the Body Image Quality of Life Inventory Scale. Body image was positively correlated with light PA (r = 0.15) and inversely correlated with BMI (Pearson’s r = −0.20) and sedentary time (r = −0.10), but not moderate PA, vigorous PA, or MVPA. Light PA and sedentary time were significantly inversely correlated (r = −0.38). When controlling for covariates, higher body image quality of life was significantly associated with higher levels of light PA (β = 0.39; *p* < 0.01) and lower sedentary time (β = −0.39; *p* = 0.02). Participants with lower body image quality of life enrolled in weight loss interventions may benefit from prescriptions of light PA in conjunction with decreasing sedentary behaviors.

## 1. Introduction

Sedentary behaviors are associated with negative health outcomes and US adults experience a high amount of time spent being sedentary [1]. Sedentary behavior refers to any waking sitting/lying behavior with low energy expenditure, rather than a lack of physical activity (PA) [2]. A recent meta-analysis found that sedentary time was associated with diabetes, cardiovascular disease, and cardiovascular all-cause mortality, independent of time spent in moderate-to-vigorous physical activity (MVPA) [3]. While achievement of MVPA is commonly promoted in weight loss interventions and public health efforts [4], light physical activity (LPA) contributes to a greater total daily energy expenditure [5] compared to MVPA. Additionally, increases in LPA have been strongly correlated with decreases in sedentary time, as up to 90% of one’s day may be spent in either sedentary time or LPA [6]. The 2018 Physical Activity Guidelines suggest decreasing all-cause mortality by replacing sedentary time with LPA [7]. LPA may include activities of daily living, such as household chores, occupational tasks, or walking slowly (less than 2.0 miles per hour) [8]. For populations needing larger calorie expenditure (e.g., adults with overweight or obesity seeking to lose weight), it is important to consider factors that may be associated with sedentary time and LPA to shift sedentary time to LPA, thereby expending more energy daily.

Young adults represent a vulnerable population in a pivotal transition time of life who are experiencing declines in PA levels, increased sedentary time, and rapid increases in obesity [9]. While declines in PA in young adults have been shown during the transition from high school to college [10] and during the college years [11], increases in sedentary time independent of PA have also been observed [12]. To date, most studies examining college students’ sedentary and PA behaviors involve self-report measurements; thus, objective device measures are necessary to better validate these findings [13]. Understanding the correlates of sedentary time and low levels of physical activity could provide direction for interventions with this population of young adults with overweight and obesity.

College students and young adults also experience high levels of psychological distress, including increased dissatisfaction with their physical appearance [14]. Young adulthood is a key risk time for body dissatisfaction and adults with excess body weight appear to be less satisfied with their bodies than their normal weight counterparts [15]. Body image is a multidimensional construct and many scales have been used to assess it, including its effect on quality of life [16]. There has been much debate whether to consider body image a stable trait or variable characteristic, thus presenting a need to quantify its effects on personal experiences and life contexts [16]. The effect of body image on quality of life has been examined in a range of health conditions, including overweight and obesity. Body image dissatisfaction may negatively impact quality of life, or contribute to psychosocial consequences including social anxiety, depression, isolation [17], and less participation in weight-related behaviors such as engagement in exercise [18].

Body dissatisfaction has been associated with physical inactivity in general populations [19] and in university students, though these data were self-reported [20]. Examinations of PA and body image have either solely focused on self-reported PA data or achievement of MVPA, which may not capture LPA levels. One assessment of objective device measures of MVPA in college students found no association between LPA and body image, though the sample was limited to normal weight females [21]. Body dissatisfaction has also been associated with increases in body weight or BMI [15], though the relationship between these variables lacks directionality due to the cross-sectional nature of evidence. In populations with overweight and obesity, body dissatisfaction may serve as both a deterrent and facilitator of PA. Those with high body dissatisfaction may have a perceived inability to lose weight and give up on healthy exercise behaviors [22]. However, contrary findings have shown that weight-concerned college women engaged in more PA during times when body dissatisfaction was higher than usual and less PA when body dissatisfaction was lower than usual [23].

The objective of this cross-sectional study was to understand how body image quality of life relates to device-measured PA and sedentary time in young adults with overweight and obesity. These relationships are complex and require additional investigation of variables that may relate to body image and moderate this relationship, such as BMI, sex, culture, and age [15]. Thus, first we examined personal characteristics associated with body image quality of life. Secondly, we assessed the relationship between average minutes of PA/day, average minutes of sedentary time/day, and body image quality of life. Finally, we examined interactions amongst variables associated with body image quality of life, average minutes of PA and sedentary time/day.

## 2. Materials and Methods

### 2.1. Study Design

This study was a baseline analysis of a parent randomized controlled trial (RCT). For details on eligibility criteria and the interventions, see Napolitano et al., 2017 [24]. In brief, the sample consisted of 459 students at one private mid-Atlantic university and one public northeastern university enrolled in a two-site, weight-loss RCT between March 2015 and February 2018. Participants were recruited through multiple university channels, such as listservs, email blasts, on-campus events, and social media posts. Interested participants completed an online survey, followed by a screening phone call to assess eligibility. Main eligibility requirements for enrolling in the RCT included (1) age 18–35 years, (2) BMI 25–45 kg/m^2^, (3) attendance at a college/university in the greater metropolitan area of each study site, (4) active Facebook users (logged in within the last month), (5) fluent in English, and (6) had regular text message access. This study was approved by the Institutional Review Board at George Washington University and informed consent was obtained from all human research participants.

### 2.2. Procedures

Eligible participants attended two in-person sessions for enrollment into the randomized controlled trial. The full description of baseline enrollment procedures is described elsewhere [24]. During the first session, objective measures of height and weight were collected by research assistants. Participants were also given an accelerometer ActiGraph GT3X+ accelerometer (Pensacola, FL) to wear for the next 7 days. Participants were instructed to wear the monitor over their right iliac crest for all waking hours of the day and to remove it only for showers, water-based activities, and sleeping. In addition, a handwritten log to track all wear-time and non-wear-time was maintained. To increase the likelihood of monitor wear-time, participants received a daily text message each morning reminding them to wear their monitor. Participants were also sent an online survey to complete within the 7 days using REDCap electronic data capture, including demographic and body image questionnaires.

After 7 days, participants returned for an additional session in which the accelerometer wear-time was validated using ActiLife software and cross-checked with their hand-written log. If participants did not meet the minimum wear-time needed, they were given the accelerometer to re-wear for the additional wear-time needed, after which they returned to the lab for verification. Research assistants verified completion of the demographic and body image questionnaires and any incomplete surveys were either completed in the research office or remotely by the participant prior to their enrollment in the study.

### 2.3. Measures

Demographics—Demographic variables included sex, age/date of birth, race, ethnicity, and school status (undergraduate or graduate).

Body Image—The impact of body image was assessed using the *Body Image Quality of Life Inventory* (BIQLI) [17]. The BIQLI is a 19-question survey and assessed the impact of feelings about physical appearance on one’s life using a 7-point Likert Scale. A higher BIQLI score has been associated with higher body satisfaction, less body shame, less of a preoccupation with being/becoming fat, less dysfunctional investment in appearance, lower body surveillance, and less internalized cultural beauty standards [17]. The BIQLI has shown test–retest reliability of 0.79 in college women [17]. In the present study, internal consistency (Cronbach’s alpha = 0.96) of the composite mean score was very high and consistent with other studies (Cronbach’s alpha = 0.94) using this tool [18].

Height, Weight, and Body Mass Index—Participants visited the clinic at baseline to obtain objective measures of height and weight. Weight and height were measured in duplicate during each check-point visit, using a digital scale (Seca Model 769) and standard portable stadiometers. Research assistants alternated between weight and height measurements. Weight was recorded to the nearest 0.2 kg, while height was recorded to the nearest 0.1 cm. Averages for both the height and weight measurements were calculated and recorded, followed by a calculation of Body Mass Index (BMI; weight (kg)/height (m^2^)) [25].

Physical Activity—ActiGraph (wGT3X-BT) accelerometers were initialized with each participant’s height, weight, sex, and date of birth using the ActiLife software. Data were collected in 60 s epochs. Data were downloaded using ActiLife software version 6.0 and wear-time was validated according to Troiano 2008 [26]. Data were included if the participant had a minimum of 4 days of 10 waking hours (600 min) of valid wear-time, similar to other studies using these assessment measures in this population [27]. Non-wear-time was defined as 60 consecutive minutes of 0 counts per minute (CPM), with allowance for 1–2 min of 0–99 CPM during this time, and was excluded from further analyses. Time spent at different intensity levels was classified using cut points identified in the Freedson Adult (1998) equation based on CPM and included light (100–1951 CPM), moderate (1952–5724 CPM), and vigorous (5725–9498 CPM) [28]. Sedentary time (h/day) was defined as accelerometer counts < 100 CPM. To account for variability in number of wear days, sedentary time (h/day) and MVPA (min/week) were calculated by multiplying the average daily total * 7. Meeting the 2018 Physical Activity Guidelines was defined as of >30 min/day of moderate-to-vigorous physical activity [7].

### 2.4. Statistical Analysis

All statistical analyses were conducted using Stata Version 13.0 (StataCorp). Participants with missing data on key study variables were excluded from the analyses. Exploratory data analysis (EDA) was used to verify that outcome measures were normally distributed and whether data transformations were warranted. EDA was also used to detect any implausible values. Initial analyses assessing variables associated with BIQLI used Pearson’s correlational analyses for continuous variables and one-way ANOVA for categorical data. All analyses with PA and sedentary time measures as the dependent variables analyzed body image as a continuous variable, with higher values representing more positive BIQLI scores. Correlational analyses were conducted to assess the relationship between BIQLI, average minutes per day of PA, and sedentary time. Simple and multiple linear regressions were run to assess the independent and adjusted effect of level of BIQLI on average minutes per day of PA and sedentary time. Multiple linear regressions included adjusting for covariates of age, sex, race, ethnicity, BMI, and school status. Interaction terms were added to the model to examine the moderating effect of variables related to those associated with BIQLI, and BIQLI and PA [29]. All statistical tests were two-sided and a *p*-value < 0.05 was considered statistically significant.

## 3. Results

The characteristics of the sample are represented in Table 1. Our sample included 404 participants (78.4% women), with a mean age of 23.3 ± 4.4 years and a mean BMI of 31.1 ± 4.4 kg/m^2^. Participants achieved an average of 219.0 ± 61.4 min of LPA/day, 44.5 ± 23.8 min of MVPA/day, and on average were sedentary for 550.4 ± 76.4 min, or 9.17 h/day.

Our first aim was to assess factors associated with body image quality of life. A one-way ANOVA showed that the effect of sex (*F*(97, 298) = 1.18, *p* = 0.14), race (*F*(97, 298) = 0.92, *p* = 0.67), and ethnicity (*F*(97, 295) = 0.95, *p* = 0.10) on BIQLI were non-significant both within and between groups. Pearson’s correlation analyses revealed no significant correlations between age and BIQLI (*r* < 0.01, *p* = 0.98), though significant inverse correlations were found between BMI and BIQLI (*r* = −0.20, *p* < 0.001).

Our second aim was to assess the relationship between average minutes of PA, sedentary time/day, and body image quality of life. Pearson’s correlation analyses revealed that average minutes of sedentary time/day were significantly inversely correlated with average minutes of LPA/day and with average minutes of sedentary time/day (see Table 2).

Due to the significant correlations in only minutes of LPA/day and BIQLI, regression analyses consisted only of LPA. A simple linear regression revealed that a more positive BIQLI was statistically significantly associated with greater average minutes of LPA/day (see Table 3). A multiple linear regression controlling for covariates indicated that a more positive BIQLI was significantly associated with increased average minutes of LPA/day. A simple linear regression revealed that a more positive BIQLI was significantly associated with decreased average minutes of sedentary time/day. A multiple linear regression controlling for covariates indicated that a more positive BIQLI was significantly associated with decreased average minutes of sedentary time/day.

Our third aim was to examine interaction effects between variables associated with body image quality of life on LPA and sedentary time. These analyses assessed the two-way interaction effects of BMI × BIQLI on LPA and sedentary time while controlling for covariates of age, race, ethnicity, and sex. For sedentary time (*F*(7, 385) = 2.51), the main effect of BIQLI on sedentary time was not significant (β = 1.13, *p* = 0.37) nor were the interaction effects of BMI and BIQLI (β = −0.05, *p* = 0.22). For LPA (*F*(7, 385) = 3.54), the main effect of BIQLI on LPA was significant (β = −2.19, *p* = 0.03), and the interaction between body image and BMI was also significant (β = 0.09, *p* < 0.01).

## 4. Discussion

This study provides data on the relationship between body image quality of life and objective device measures of physical activity and sedentary time in young adults with overweight and obesity. Overall, the results from this study indicate that sedentary time had a strong inverse correlation with LPA, and that body image quality of life was positively associated with LPA and inversely associated with sedentary time. Controlling for covariates such as BMI and sex that may confound the relationship between body image quality of life, PA, and sedentary time, we still found those relationships remained significant. This study extends existing evidence using objective measures of sedentary time and PA while providing insight into their relationship with body image quality of life in young adults with overweight and obesity.

We found that a more positive body image quality of life was positively correlated with LPA, though no differences in other PA intensities were found. An unexpected finding was the lack of relationships at intensities other than LPA. This may be due in part to the complex relationship between body image, BMI, and PA, as findings in the literature have been contradictory. Previous research has documented positive associations between body image and MVPA, though no significant differences between body image and LPA in normal weight college students [21]. Longitudinal findings from college women with overweight showed an inverse relationship between PA and body satisfaction; however, the measurement of PA was limited to step counts/day [23]. Our results provide further insight into PA intensity levels and sedentary behaviors in a similar population, though the present study differs in that there was no relationship between body image and MVPA. It has been suggested that individuals with weight concerns may engage in greater PA levels due to an outcome expectation of weight loss, though consideration has not been given to outcome expectations for engaging in sedentary behaviors and its effect on PA [30]. It is possible that those with body dissatisfaction do not perceive health benefits, increasing caloric expenditure, and weight loss from decreasing sedentary behaviors. Our findings and others show greater sedentary time is associated with lower levels of LPA [6]. Thus, those with body dissatisfaction may experience increased sedentary behaviors and lower levels of LPA, though similar levels of MVPA, as compared to those with greater body satisfaction.

An additional purpose of this study was to assess variables associated with body image quality of life and further examine the moderating effect on PA and sedentary time. The assessment of variables associated with body image revealed BMI was statistically significantly correlated with body image, which is similar to previous findings in young adults [31]. Our analyses examining interactions of BMI with body image on LPA and sedentary time suggest BMI may have a moderating effect on the relationship between body image and LPA, though not body image and sedentary time. Previous research examining these variables has mainly utilized self-report measures of MVPA, and findings indicate that adults who were dissatisfied with their bodies were less likely to engage in MVPA, irrespective of actual weight status [32]. It has been suggested that those with body dissatisfaction may engage in MVPA as a means of weight control, though this may not translate to engaging in LPA. For sedentary time, we found BMI did not moderate the effect of body image and suggests those with body dissatisfaction are spending increasingly greater amounts of time being sedentary irrespective of weight status. In addition to BMI, it is also important to examine body discrepancy, or the difference between the self-perception of the body and its actual shape [33]. Prior work shows higher BMI scores were correlated with increased body discrepancy [34]. Thus, future studies need to also report on the BMI to self-perception discrepancy so we can better understand differences, particularly differences among diverse populations.

In this population of young adults, 67% (n = 269) of the sample met MVPA guidelines of achieving at least 30 min of MVPA/day, which is well above the national average. While promising, previous studies have shown total waking hours spent in MVPA range from 4% in adults [35] to 10% in young adults [36], with most waking hours spent in either LPA or sedentary time. Previous research has shown no association between MVPA and LPA [3], though strong inverse correlations between LPA and sedentary time exist. The 2018 Physical Activity Guidelines Advisory Committee Scientific Report has identified insufficient amounts of evidence to understand the effect of LPA on weight attenuation and all-cause mortality in light of the relationship between LPA and sedentary time [7]. However, researchers have found that independent of all PA levels, sedentary time greater than 4–8 h increases one’s all-cause mortality risk by 2%, and greater than 8 h/day increases one’s risk by 8%. Independent of PA, our population surpasses the 8 h cut point (mean time = 9.26 h), placing them at a >8% risk of all-cause mortality. The 2018 Physical Activity Guidelines Scientific Report has also identified there is insufficient evidence to determine whether the association between sedentary time and all-cause mortality is affected by LPA or MVPA. Considering these insufficient findings, a call for future research examining the health effects of replacing sedentary time with varying levels of PA has been issued [7]. Our findings substantiate further examination of the relationship between LPA and sedentary time and how they relate to weight attenuation and all-cause mortality, particularly in those with body dissatisfaction.

Results from this study have several practical implications. Increases in LPA have been associated with increases in total energy expenditure, resistance to fat gain [37], and reductions in sedentary time; thus, the benefits of LPA are numerous. Adverse health effects are highly associated with decreased levels of LPA and increased sedentary time [38] and preliminary evidence on improvement in cardiometabolic health associated with increases in LPA is promising. Thus, researchers designing interventions and practitioners prescribing physical activity focused on increasing LPA and decreasing sedentary behaviors in the long term may have the potential to positively impact health outcomes. Weight loss interventions focusing solely on increasing MVPA or resistance training may target appearance-related outcomes, which may be detrimental to engaging in PA long term [39]. These methods may not be salient for persons with body dissatisfaction, as they may deter one from engaging in activities that are less intense than achieving the standard MVPA guidelines, possibly inducing further sedentary time. Researchers may investigate body dissatisfaction screening as a means of tailoring activity prescriptions in an effort to decrease sedentary time, increase activity levels, and positively impact health outcomes.

There are several limitations that must be identified. First, these results can only be generalized to a population with overweight or obesity seeking an intervention to help them lose weight or attain a healthy body weight. This may have affected the motivations participants had for engaging in physical activity. Second, this study only assessed one dimension of body image (how body image affected quality of life), and other body image measures may produce differing results. Third, it has been well-documented that reducing energy intake is needed to induce weight loss [40]. The focus of this study was on light activity and sedentary behavior. However, it is recognized that dietary intake is an important factor that is associated with body weight, energy for physical activity, and potentially body image. Thus, diet composition presents another area for future studies to examine. Fourth, while accelerometers have become the gold standard in assessing objective device measures of PA, monitors worn at the hip have been shown to under detect upper body movement, load carriage, or changes in surface or terrain. These movements could occur during common household chores/activities and may cause discrepancies in counts, thus misclassifying and underreporting light PA. In addition, it is a measure of the duration of time spent in sedentary time, LPA, and MVPA while the device was worn rather than capturing all 24 h of activity. However, this study is strengthened by the use of objective device measures of PA, as many researchers have relied on self-report measures to examine its relationship to body image [19].

## 5. Conclusions

In this population of young adults with overweight and obesity, a more positive body image quality of life was associated with increased average minutes of light physical activity and decreased average minutes of sedentary time per day. We found that an interaction between body image and BMI explained additional variance in minutes of LPA/day. The finding that LPA is affected by the interaction between body image and BMI has implications for future physical activity recommendations and programming. Public health and clinical efforts to address activity among those with overweight and obesity should account for the complex relationships between body image, BMI, physical activity, and sedentary time. Our results contribute to previous evidence suggesting the complex interplay between body image and PA, while contributing new findings regarding LPA and sedentary time to the literature. Future research is needed to investigate the effectiveness of improving cardiometabolic health by promoting LPA either in lieu of or concurrently with standard MVPA guidelines in populations with overweight and obesity at risk for developing poor body image quality of life.

## Figures and Tables

**Table 1:** Participant demographics (N = 404).

Variable	Mean ± SD or n (%)
Age, years	23.3 ± 4.4
Gender	
Female	317 (78.4)
Male	87 (21.5)
BMI, kg/m^2^	31.1 ± 4.4
Race ˆ	
Asian	36 (8.9)
Black or African American	82 (20.3)
Native Hawaiian	2 (0.5)
White	224 (55.4)
Multi-racial	18 (4.5)
Unknown/Prefer not to answer	42 (10.4)
Ethnicity ˆ	
Hispanic/Latino	57 (14.1)
Not Hispanic/Latino	332 (82.2)
Unknown/Prefer not to answer	15 (3.7)
School status	
Undergraduate	231 (57.2)
Graduate	173 (42.8)
Body Image Quality of Life Inventory score *	57.0 ± 23.3
Light PA/day (average minutes)	219.0 ± 61.4
Moderate PA/day (average minutes)	41.8 ± 20.7
Vigorous PA/day (average minutes)	4.0 ± 7.5
MVPA/day (average minutes)	44.5 ± 23.8
Sedentary time/day (average minutes)	550.4 ± 76.4

^ Optional response, thus missing data are not included in these totals. * Range 0–114. Higher score = more positive body image quality of life.

**Table 2:** Bivariate correlations between body image quality of life score, physical activity intensity levels, and sedentary time (average minutes/day).

Variable	1	2	3	4	5	6
BIQLI Score	-					
Average Minutes of Physical Activity/Day						
**2** Light/day	0.15 *	-				
**3** Moderate/day	0.02	0.06	-			
**4** Vigorous/day	0.09	−0.06	0.34 *	-		
**5** Moderate-to-vigorous/day	0.04	0.03	0.96 *	0.57 *	-	
Average Minutes of Sedentary/day	−0.11 ^	−0.38 *	−0.26 *	−0.06	−0.24 *	-

* Significant at <0.01; ^ significant at <0.05.

**Table 3:** Simple and multiple linear regressions examining effects of body image on average minutes of light PA/day and sedentary time/day.

Variable	*R* ^2^	*β*	*SE*	*t*	*p*	*CI*
Light PA/day	0.04	0.44	0.13	3.28	0.002	0.18 to 0.70
Light PA/day (adjusted) *	0.02	0.39	0.13	2.95	<0.001	0.13 to 0.65
Sedentary time/day	0.01	−0.34	0.16	−2.08	0.04	−0.07 to −0.02
Sedentary time/day (adjusted) *	0.04	−0.39	0.17	−2.34	0.02	−0.73 to −0.06

* Model adjusted for age, sex, race, ethnicity, school status, and BMI.

## Data Availability

The data presented in this study are available on request from the corresponding author.
